# Supplementary material for: Tissue-specific transcriptomes reveal potential mechanisms of microbiome heterogeneity in an ancient fish
Source: Database (Oxford). 2023 Aug 17;2023:baad055. doi: 10.1093/database/baad055 (PMC10434735; doi:10.1093/database/baad055)

Supplementary Figure S1. Uniqueness of gene sets among all transcriptomes, including the overall and tissue-specific transcriptomes as visualized with UpSetR. While 3,065 genes are uniquely present in the overall transcriptome, measures of uniqueness for the tissue-specific transcriptome were biased by inclusion of the overall transcriptome that was made with data used to assemble all of the others. Thus any otherwise unique gene or gene set would then be shared between the overall transcriptome and the tissue-specific transcriptome. Therefore, analyses of uniqueness among tissues focused on the set of the tissue-specific transcriptomes.


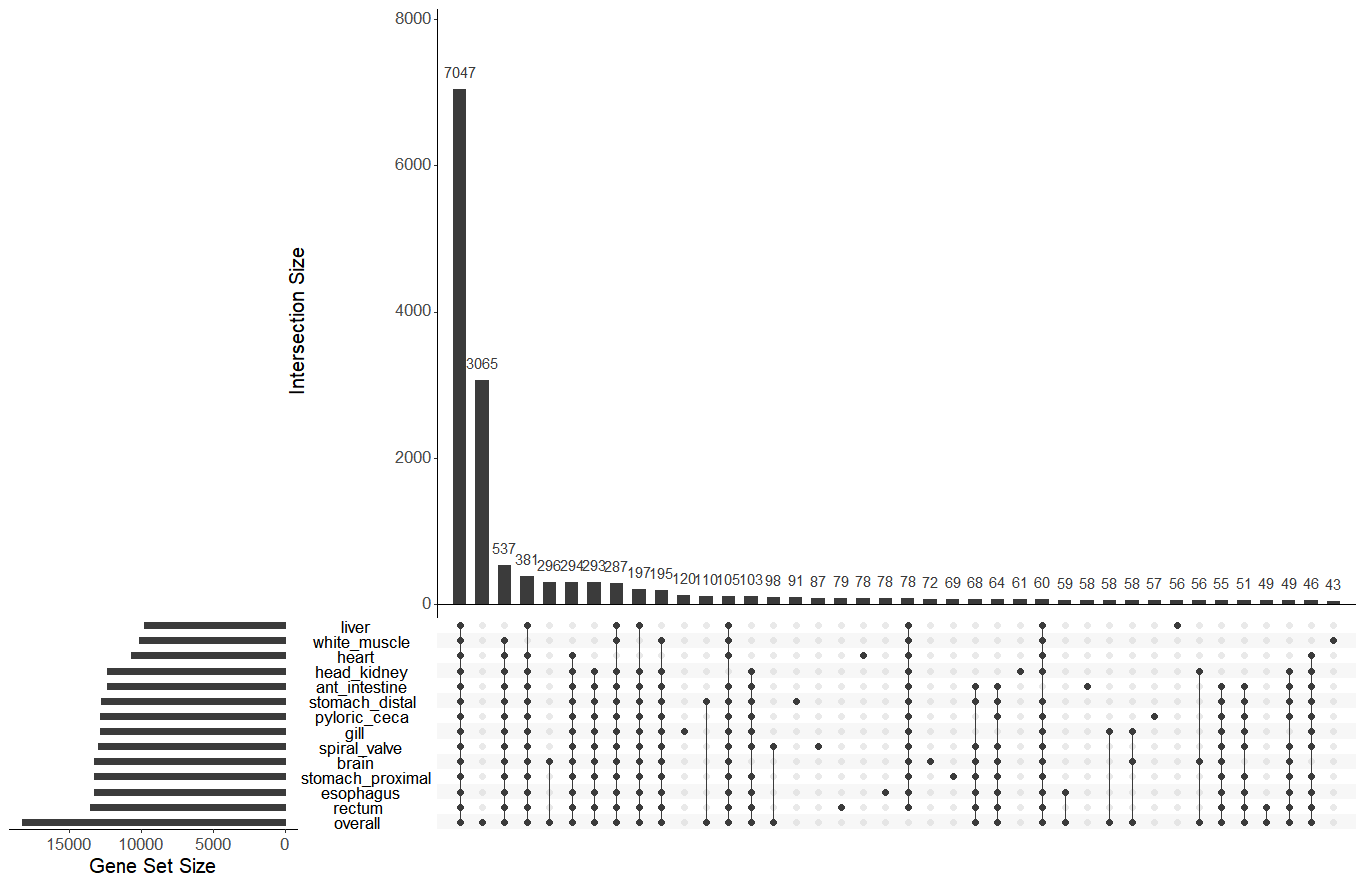


Supplementary Figure S2. Uniqueness of gene ontology terms from the Molecular Function 2021 database among 13 tissue-specific transcriptomes, as assessed with EnrichR and visualized with UpSetR.


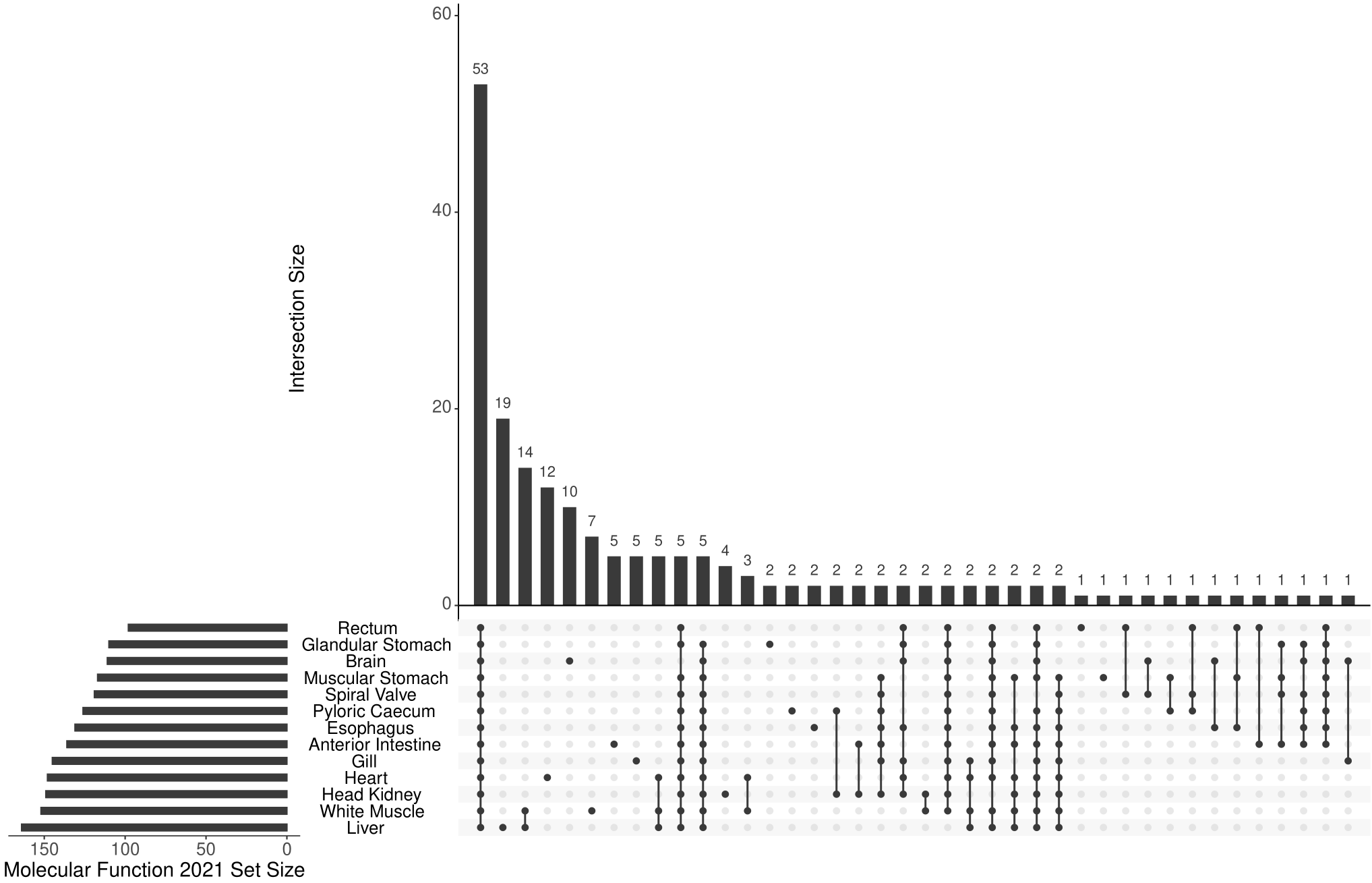


Supplementary Figure S3. Uniqueness of gene ontology terms from the Cellular Component 2021 database among 13 tissue-specific transcriptomes, as assessed with EnrichR and visualized with UpSetR.


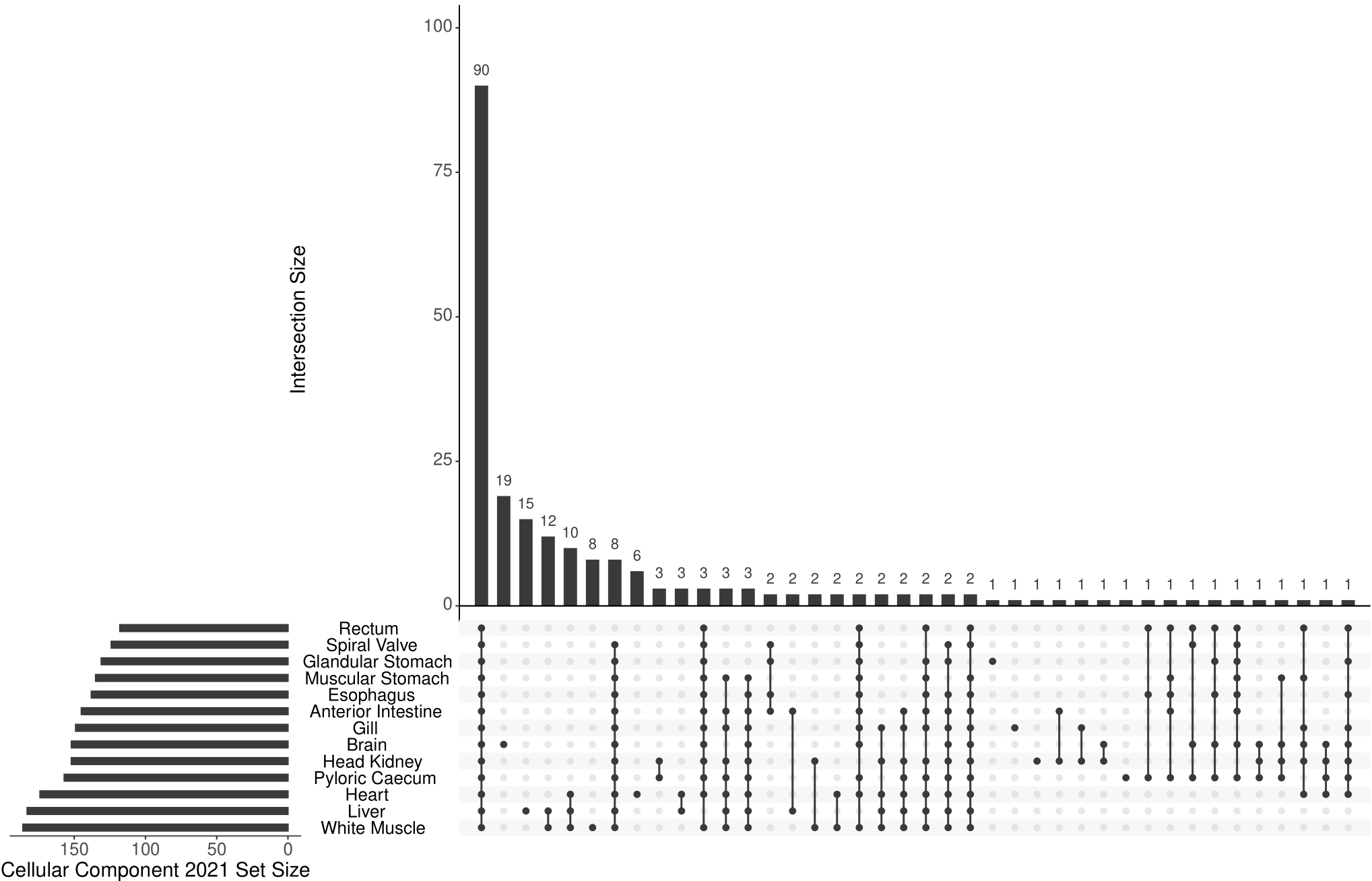


Supplementary Figure S4. Uniqueness of gene ontology terms from the Biological Process 2021 database among 7 gut tissue-specific transcriptomes, as assessed with EnrichR and visualized with UpSetR.


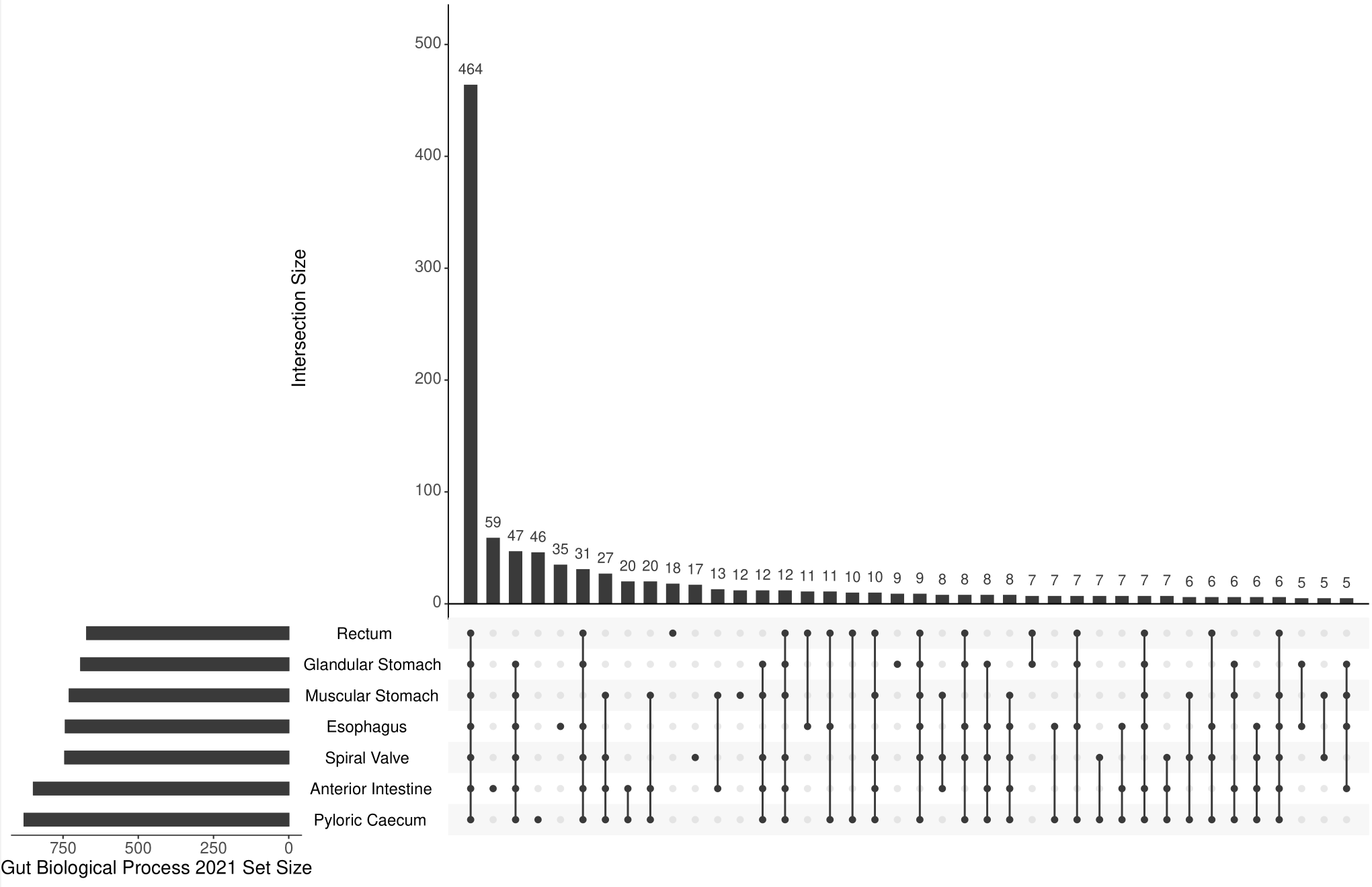


Supplementary Figure S5. Principal components analyses of present gene ontology terms among 13 tissue-specific transcriptomes as assessed with EnrichR and visualized with UpSetR. A) represents an analysis of terms from the biological process 2021 database, B) an analysis of terms from the molecular function 2021 database, and C) an analysis of terms from the cellular component 2021 database.


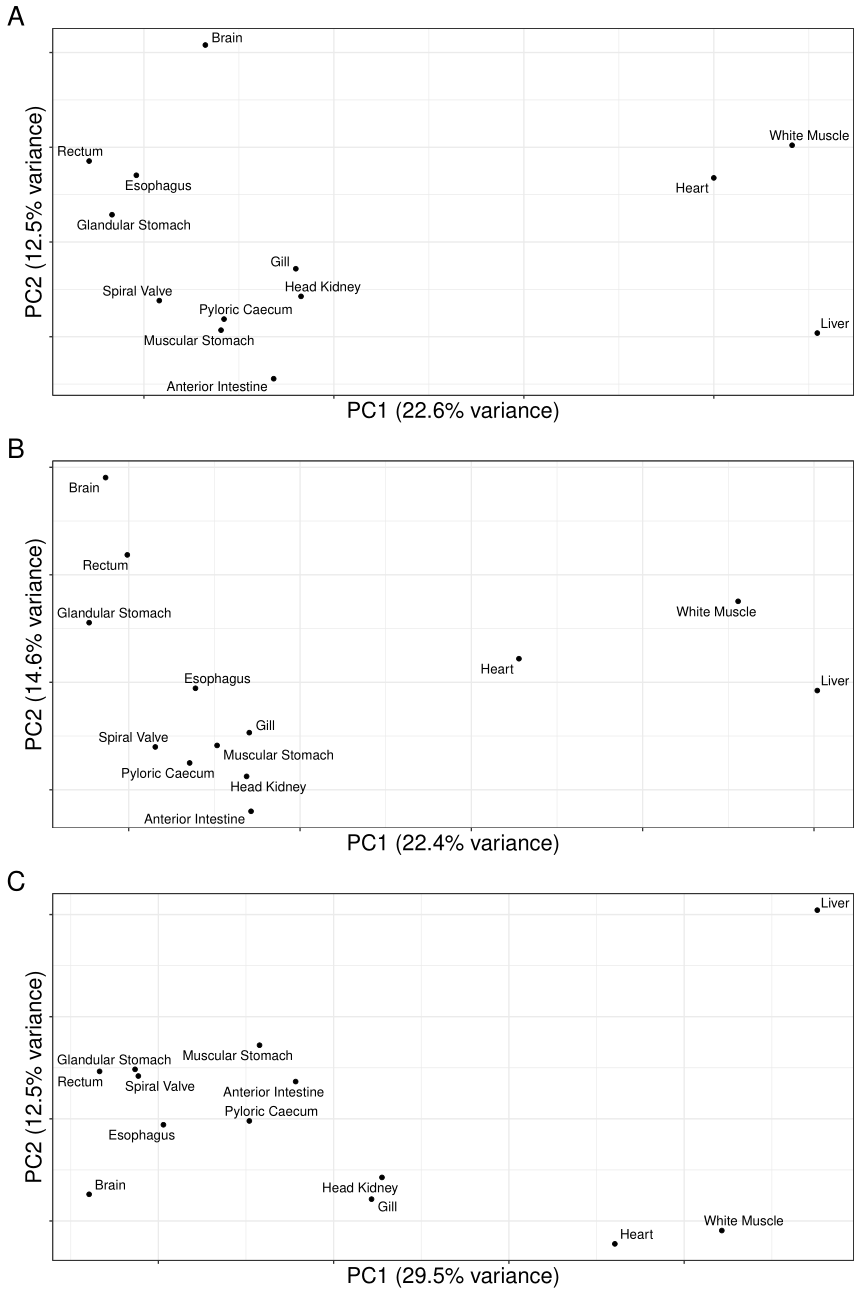


Supplementary Figure S6. Uniqueness of microbial (bacteria, viruses, and archaea) genera among 13 tissue-specific transcriptomes as visuzalized by UpSetR.
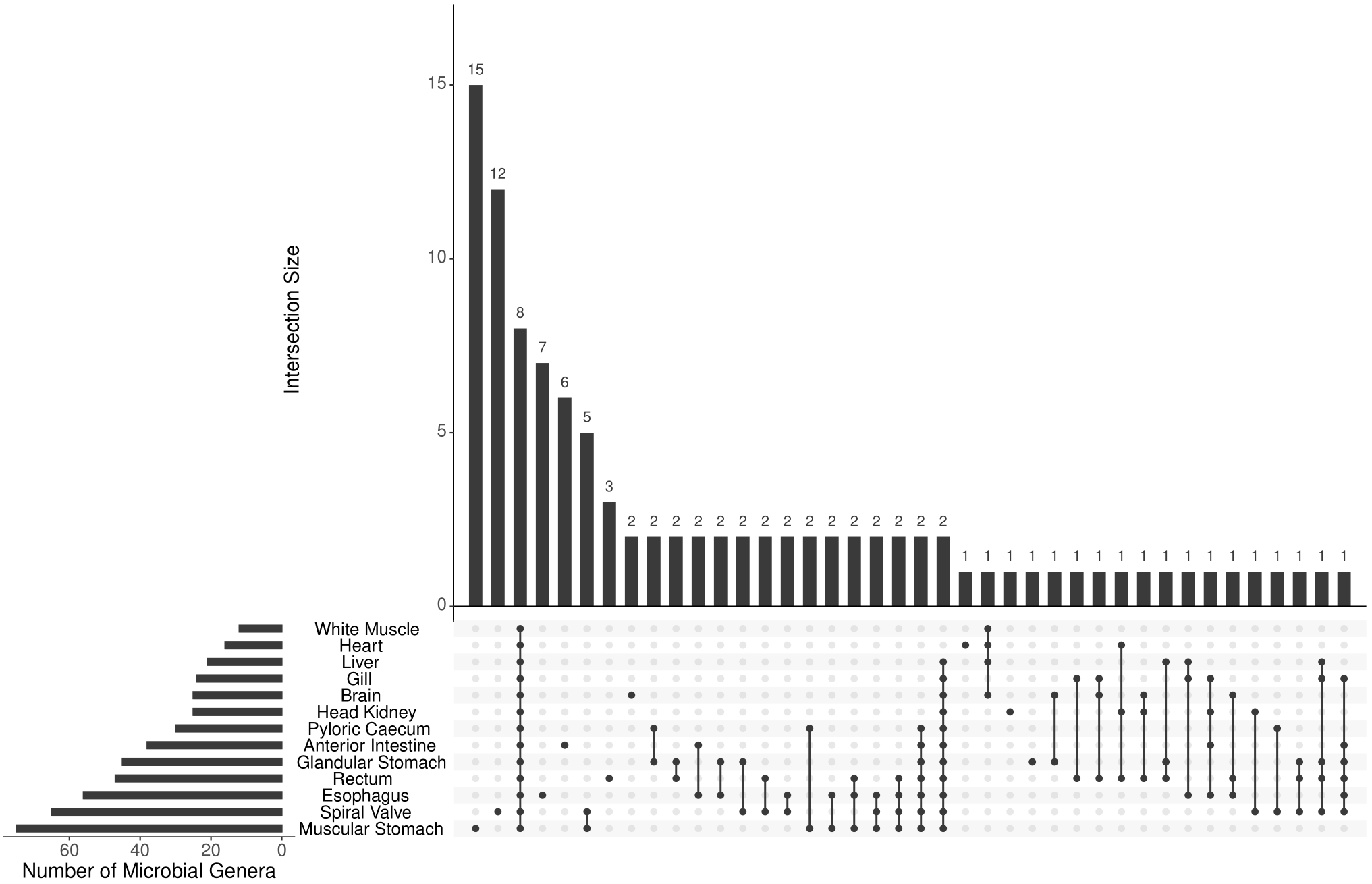

Supplement: baad055_Supp [file baad055_supp.zip › suppl_data/LS_transcriptome_supplementary_figures_Apr10...docx]
